# Electronic Performance Monitoring in the Digital Workplace: Conceptualization, Review of Effects and Moderators, and Future Research Opportunities

**DOI:** 10.3389/fpsyg.2021.633031

**Published:** 2021-05-21

**Authors:** Thomas Kalischko, René Riedl

**Affiliations:** ^1^Digital Business, School of Business and Management, University of Applied Sciences Upper Austria, Steyr, Austria; ^2^Institute of Business Informatics – Information Engineering, Johannes Kepler University Linz, Linz, Austria

**Keywords:** computer monitoring, electronic performance monitoring, stress, workplace surveillance, human-media interaction, technostress, home office, review

## Abstract

The rise of digital and interconnected technology within the workplace, including programs that facilitate monitoring and surveillance of employees is unstoppable. The COVID-19-induced lockdowns and the resulting increase in home office adoption even increased this trend. Apart from major benefits that may come along with such information and communication technologies (e.g., productivity increases, better resource planning, and increased worker safety), they also enable comprehensive Electronic Performance Monitoring (EPM) which may also have negative effects (e.g., increased stress and a reduction in job satisfaction). This conceptual article investigates EPM to better understand the development, adoption, and impact of EPM systems in organizations. The EPM literature published since the 1980s constitutes the basis for this conceptual article. We present a framework which is intended to serve as foundation for future studies. Moreover, we reviewed more than three decades of empirical EPM research and identified six major outcomes that are influenced by the use of an EPM system, as well as a large number of moderator variables. Based on our conceptual analyses and the resulting insights, which also include privacy, ethical, and cultural considerations, we discuss future research opportunities where we also refer to design implications for EPM systems.

## Introduction

The term “Electronic Performance Monitoring” (hereafter EPM) has its origin in the term “Electronic Work Monitoring,” which was introduced at the U.S. Congress, Office of Technology Assessment, 1987. The original term “Electronic Work Monitoring” refers to the “computerized collection, storage, analysis, and reporting of information about employees’ productive activities” (U.S. Congress, Office of Technology Assessment, 1987, p. 27), and this original conceptualization has been frequently used as a definition of EPM in the scientific literature over the past decades. This and related definitions (e.g., Grant and Higgins, 1989) mainly referred to the context of call centers in which performance monitoring was already an existing practice several decades before. However, early in the 1990s it became clear that EPM also plays a notable role in other industries. Corlett (1992), referring to the American engineer Frederick W. Taylor (1856-1915) and his concept of “industrial efficiency,” tellingly stated that the trend of EPM can lead to the “Taylorization of the office.” A major implication of this reference to Taylor was that the surveillance aspect of EPM along with a relationship of distrust between the organization (supervisor) and employee became the dominant perspective. What follows is that EPM use in organizations was typically perceived negatively, in particular with adverse consequences for employees.

Today EPM is a widespread practice in work environments. Managers frequently have access to their employees’ performance data, as well as other data on behavior which allows them to check, among other things, the working pace, the degree of work accuracy, log-in and log-off times, and even the number and duration of breaks (Aiello and Kolb, 1995; Oz et al., 1999; The Guardian, 2018). Importantly, EPM may lead to advantages, such as higher productivity, better resource planning, or increased staff safety (Martin and Freeman, 2003). However, disadvantages such as lower employee morale and satisfaction, as well as stress and the development of stress-related illnesses are also reported in the scientific literature (Aiello and Svec, 1993; George, 1996; Jeske and Kapasi, 2017).

Since the introduction of EPM, technology in general and also technology that enables computer-based monitoring has advanced rapidly. In a recent paper, Edwards et al. (2018) summarize the historical development of employee surveillance from a technology perspective. In essence, they introduce several stages of surveillance: Surveillance 1.0 stands for extensive analog monitoring, Surveillance 2.0 for the recording of keyboard activities, application usage and mouse clicks, and Surveillance 3.0 for the tracking of emails and website activity, resulting in access to data on “personal relationships, thoughts, opinions, preferences and interactions” (p. 5). In the recent past, ubiquitous computing and the Internet of Things (IoT) have made possible “real-time, ubiquitous and unobtrusive surveillance of employees […] by small cheap sensor technology capable of being embedded within the working environment” (p. 6), referred to as Surveillance 4.0. Today, we increasingly observe Surveillance 5.0, or the age of algorithms, in which “data analytics algorithms are designed to generally spot patterns in large amounts of data, enabling categorization and profiling [… enabling] automated or assisted decision making about hiring, firing and internal promotion or disciplining” (p. 6). Machine learning algorithms, big data, and artificial intelligence constitute the technological basis for this most advanced form of monitoring (Wenzel and van Quaquebeke, 2017; Yanqing et al., 2019).

Against the background of these most recent technological developments it is not surprising that EPM is experiencing a heyday. *The Guardian (2015)* characterized EPM as a “digital panopticon” which started off by monitoring emails and phone calls, and now even includes tracking of text messages, screenshots, keystrokes, social media activity, private messaging services, and face-to-face interactions with colleagues. In a more recent article, *The Guardian (2018)* further reported that EPM has become a significant privacy issue in particular in tech companies, but also in other industries. Similar reports can be found in many other magazines and newspapers including the German outlet *Süddeutsche Zeitung (2019)*, which reported on the monitoring methods of Zalando, and *The New York Times (2017)*, which reported on a company implanting microchips in employees.

In 2019, the market research firm Gartner provided an outlook into the future of the employee monitoring market for 2020 (Gartner, 2019). They estimated that almost 80% of all companies will be using monitoring software to keep track of their organizational goals and their employees. In 2018, this value was 50% and hence a dramatic increase in the application of EPM can be observed. In 2019, the market research firm Accenture reported that 62% of surveyed C-level executives “said that their organizations are using new technologies to collect data on their people and their work to gain more actionable insights – from the quality of work and the way people collaborate to their safety and well-being – fewer than one-third (30%) are very confident that they are using the data responsibly” (Accenture, 2019). In 2020, as a consequence of the increasing home office adoption due to the COVID-19 pandemic, organizations have even increased the level of computer-based monitoring. Recent newspaper reports such as those by the BBC (2020) or Businessinsider (2020) indicate new ways of surveillance during COVID-19-induced home office hours. The MIT Technology Review (2020) confirms these developments. As another example, *The Guardian (2020)* writes: “Microsoft has been criticized for enabling ‘workplace surveillance’ after privacy campaigners warned that the company’s ‘productivity score’ feature allows managers to use Microsoft 365 to track their employees’ activity at an individual level” (note that Microsoft scaled back the corresponding features recently, Morse, 2020). Altogether, as documented in many articles, today EPM is predominantly perceived as a significant issue in the economy and in society that deserves attention. Fueled by the current COVID-19 pandemic and the resulting desire of employers to also keep track of employees’ activities in their home offices, EPM and the resulting technology-mediated surveillance practices have become an even more important topic in society today.

Considering this call for attention, we thought it might be useful to concisely document and systemize major insights from the academic literature on EPM and to integrate this empirical basis. Specifically, this article makes the following contributions. First, we developed a research framework to provide a conceptual basis on a critical topic from an abstracted point of view (summarized in Figure 2). Second, we reviewed the academic literature to structure what is known about EPM, particularly with respect to outcomes and moderators (summarized in Table 1 and Figure 3). Third, we reflect on important privacy, ethical, and cultural considerations. Fourth and finally, we outline opportunities to guide future research, including possible avenues for EPM design science research.

**TABLE 1:** Research findings on EPM use and outcome variables along with moderator effects.

**Stress**	Aiello and Kolb (1995) [−]; Bartels and Nordstrom (2012) [∼]; Carayon (1994) [−]; Davidson and Henderson (2000) [∼]; DiTecco et al. (1992) [∼]; Galletta and Grant (1995) [∼]; Hawk (1994) [−]; Henderson et al. (1998) [−]; Huston et al. (1993) [−][+]; Kolb and Aiello (1996) [−]; Mallo et al. (2007) [−]; Nebeker and Tatum (1993) [∼]; Rogers et al. (1990) [−]; Sarpong and Rees (2014) [+]; Smith et al. (1992) [−]; Sprigg and Jackson (2006) [−]; Varca (2006) [−]; Visser and Rothmann (2008) [−]; Westin (1992) [−] Number of studies reporting a positive effect: 2 Number of studies reporting a negative effect: 13 Number of studies reporting no effect: 5
Moderators	Moderators increasing the EPM effect on stress: High Age: Mallo et al. (2007) [↑]; High Level of Monitoring: Aiello and Kolb (1995) [↑]; High Task Difficulty: Davidson and Henderson (2000) [↑]; Nebeker and Tatum (1993) [↑]; Locus of Control: Kolb and Aiello (1996) [↑] Moderators decreasing the EPM effect on stress: Comprehensive Announcement: Aiello and Kolb (1995) [↓]; Bartels and Nordstrom (2012) [↓]; Henderson et al. (1998) [↓]; Huston et al. (1993) [↓]; Mallo et al. (2007) [↓]; Rational Explanation: Bartels and Nordstrom (2012) [↓]
Example	A sales worker perceives stress because the supervisor can monitor daily activities (e.g., number of visits to potential clients, number of sent offers) in a customer relationship management system.

**Motivation**	Aiello and Kolb (1995) [+]; Arnaud and Chandon (2013) [−]; Bartels and Nordstrom (2012) [+]; Gichuhi et al. (2016) [+]; O’Donnell et al. (2013) [−]; Rietzschel et al. (2014) [∼] Number of studies reporting a positive effect: 3 Number of studies reporting a negative effect: 2 Number of studies reporting no effect: 1
Moderators	Moderators increasing the EPM effect on motivation: Comprehensive Announcement: Aiello and Kolb (1995) [↑]; Bartels and Nordstrom (2012) [↑]; Rational Explanation: Bartels and Nordstrom (2012) [↑] Moderators decreasing the EPM effect on motivation: High Level of Monitoring: O’Donnell et al. (2013) [↓]; Rietzschel et al. (2014) [↓]; Low Personal Need for Structure: Rietzschel et al. (2014) [↓]
Example	An employee is more motivated because the document management system visualized that he is a highly active person with respect to editing documents. Before this system was implemented, it was difficult for the supervisor to distinguish the more active from the less active employees.

**Job Satisfaction**	Bartels and Nordstrom (2012) [∼]; Chalykoff and Kochan (1989) [+]; Douthitt and Aiello (2001) [−]; Holman et al. (2002) [−]; Jeske and Santuzzi (2015) [−]; McNall and Stanton (2011) [+]; Nebeker and Tatum (1993) [∼]; Rietzschel et al. (2014) [∼]; Stanton and Julian (2002) [+]; Wells et al. (2007) [+]; Zweig and Scott (2007) [+] Number of studies reporting a positive effect: 5 Number of studies reporting a negative effect: 3 Number of studies reporting no effect: 3
Moderators	Moderators increasing the EPM effect on job satisfaction: Comprehensive Announcement: Bartels and Nordstrom (2012) [↑]; Nebeker and Tatum (1993) [↑]; Stanton and Julian (2002) [↑]; High Job Control: Holman et al. (2002) [↑]; Rational Explanation: Bartels and Nordstrom (2012) [↑]; Wells et al. (2007) [↑]; Rewards: Nebeker and Tatum (1993) [↑]; Supervisor Support: Douthitt and Aiello (2001) [↑]; Holman et al. (2002) [↑]; Nebeker and Tatum (1993) [↑] Moderators decreasing the EPM effect on job satisfaction: High Level of Monitoring: Jeske and Santuzzi (2015) [↓]; Rietzschel et al. (2014) [↓]; Low Perceived Control: Jeske and Santuzzi (2015) [↓]; Low Personal Need for Structure: Rietzschel et al. (2014) [↓]; Privacy Invasion: McNall and Stanton (2011) [↓]
Example	A sales representative has a low degree of job satisfaction because his smartphone which was provided by the organization allows for determination of location (based on GPS technology).

**Trust**	Alder et al. (2006) [+]; Alge et al. (2004) [−]; Carpenter et al. (2016) [∼]; Holland et al. (2015) [−]; Hovorka-Mead et al. (2002) [+]; Jensen and Raver (2012) [−]; McNall and Roch (2009) [+]; Stanton and Sarkar-Barney (2003) [−]; Westin (1992) [−] Number of studies reporting a positive effect: 3 Number of studies reporting a negative effect: 5 Number of studies reporting no effect: 1
Moderators	Moderators increasing the EPM effect on trust: Comprehensive Announcement: Alder et al. (2006) [↑]; Supervisor Support: Alder et al. (2006) [↑] Moderators decreasing the EPM effect on trust: Manual Job Type: Holland et al. (2015) [↓]
Example	A software developer feels distrust of the supervisor because he analyses the number of daily programmed lines of code and the number of instant messages exchanged with other developers in order to infer productivity.

**Commitment**	Bhave (2014) [∼]; Chang et al. (2015) [−]; Greenberg and Barling (1999) [−]; Jensen and Raver (2012) [∼][−]; Jeske and Santuzzi (2015) [−]; Martin et al. (2016) [−]; Niehoff and Moorman (1993) [∼]; O’Donnell et al. (2013) [−]; Sherif and Al-Hitmi (2017) [∼]; Spitzmüller and Stanton (2006) [−]; Vries and van Gelder (2015) [+]; Wellen et al. (2009) [−]; Yost et al. (2019) [−] Number of studies reporting a positive effect: 1 Number of studies reporting a negative effect: 9 Number of studies reporting no effect: 4
Moderators	Moderators increasing the EPM effect on commitment: Competition: Sherif and Al-Hitmi (2017) [↑]; High Technology Experience: Spitzmüller and Stanton (2006) [↑]; Paradoxical Leadership: Sherif and Al-Hitmi (2017) [↑] Moderators decreasing the EPM effect on commitment: High Level of Monitoring: Jeske and Santuzzi (2015) [↓]; Martin et al. (2016) [↓]; O’Donnell et al. (2013) [↓]; Wellen et al. (2009) [↓]; Low Perceived Control: Jeske and Santuzzi (2015) [↓]; Negative Attitude toward EPM: Martin et al. (2016) [↓]; Privacy Invasion: Yost et al. (2019) [↓]
Example	An employee feels little commitment to this organization since he learned that a software tool takes pictures of the screen every 10 min during videoconferences.

**Performance**	Aiello and Svec (1993) [−]; Aiello and Kolb (1995) [∼]; Al-Rjoub et al. (2008) [∼]; Bartels and Nordstrom (2012) [+]; Becker and Marique (2014) [−]; Davidson and Henderson (2000) [∼]; Douthitt and Aiello (2001) [−]; Goomas and Ludwig (2009) [+]; Griffith (1993) [∼]; Henderson et al. (1998) [+]; Huston et al. (1993) [+]; Irving et al. (1986) [+]; Kolb and Aiello (1996) [∼]; Larson and Callahan (1990) [+]; Ludwig and Goomas (2009) [+]; Mallo et al. (2007) [−]; Nebeker and Tatum (1993) [+]; O’Donnell et al. (2013) [+]; Stanton and Barnes-Farrell (1996) [∼]; Stanton and Sarkar-Barney (2003) [∼] Number of studies reporting a positive effect: 9 Number of studies reporting a negative effect: 4 Number of studies reporting no effect: 7
Moderators	Moderators increasing the EPM effect on performance: Comprehensive Announcement: Bartels and Nordstrom (2012) [↑]; Nebeker and Tatum (1993) [↑]; Consequences: Larson and Callahan (1990) [↑]; Performance Feedback: Goomas and Ludwig (2009) [↑]; Ludwig and Goomas (2009) [↑]; Nebeker and Tatum (1993) [↑]; Rational Explanation: Bartels and Nordstrom (2012) [↑]; Moderators decreasing the EPM effect on performance: High Age: Mallo et al. (2007) [↓]; High Level of Monitoring: Aiello and Svec (1993) [↓]; O’Donnell et al. (2013) [↓]; High Task Difficulty: Becker and Marique (2014) [↓]; Davidson and Henderson (2000) [↓]; Huston et al. (1993) [↓]; Larson and Callahan (1990) [↓]; Mallo et al. (2007) [↓]; Nebeker and Tatum (1993) [↓]; O’Donnell et al. (2013) [↓]
Example	The performance of a bank employee increased since he heard that the company uses data from the workflow management system for process mining purposes (that have the goal, among others, to identify long handling times, e.g., in loan processing).

*The application of EPM may have a positive [+], negative [−], or no effect [∼] on an outcome variable. Two square brackets indicate two studies with different results. Moderation effects are illustrated with “↑” or “↓” symbols, indicating an increasing or decreasing effect on the outcome variable.*

Because EPM research is fragmented and has been published in outlets across various scientific disciplines, including Information Systems (e.g., George, 1996), Psychology (e.g., Aiello and Kolb, 1995), and Human-Computer Interaction (e.g., Carayon, 1994), a documentation of the existing knowledge that could instigate a more cumulative research tradition in the future is even more critical than in less fragmented domains. Another motivation why we investigate EPM now is that the topic lost much of its relevance after the 1990s, but suddenly became relevant again due to technological developments in the very recent past (Edwards et al., 2018).

## Methodology

**FIGURE 1:**
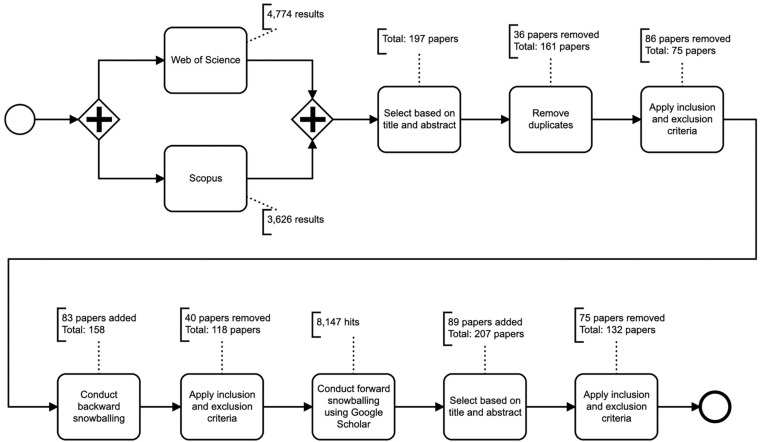
Overview of the search process.

In order to review several decades of EPM research, we conducted a literature search for journal publications that have been published since the term “Electronic Work Monitoring” was coined at the U.S. Congress, Office of Technology Assessment, 1987 (the term “Electronic Performance Monitoring” became more prominent within the scientific community over time). The literature review was conducted based on existing methodological recommendations (Webster and Watson, 2002; Kitchenham and Charters, 2007; vom Brocke et al., 2009). Based on primary selected papers, we conducted an initial review, followed by backward snowballing, a second review of the associated results, and a subsequent forward snowballing. Figure 1 summarizes our search process.

### Search Strategy

The keywords used for the literature research were mainly derived from landmark publications that offer a broad introduction into the field of electronic performance monitoring such as Stanton (2000), Ball (2010), and Ravid et al. (2020). We used search terms that are representative for the EPM literature, namely “electronic performance monitoring,” “electronic monitoring,” “EPM,” “workplace monitoring,” and “workplace surveillance.” We set the focus of our search on only peer-reviewed journal articles within the field of business, computer science, human resource, psychology, and social sciences. We searched within the databases Scopus and Web of Science. This search process was conducted from 09/01/2020 to 09/10/2020 and encompasses publications from 1987 to 2020. This method resulted in 8,400 hits. Subsequent to the initial search we applied a filtering strategy (cf. Section “Filtering Strategy”) followed by backward snowballing, another review and forward snowballing (cf. Section “Backward and Forward Snowballing”).

### Filtering Strategy

Within the first step we removed all unrelated papers based on reading title and abstract which resulted in 197 papers. After removing duplicates, the result yielded in 161 unique papers. Those remaining papers were then analyzed in detail and the following inclusion and exclusion criteria were applied:

*Inclusion Criterion:* The article focuses on electronic performance monitoring and/or investigates determinants that interact with EPM, and/or EPM related outcomes.

*Exclusion Criterion:* Papers focusing on different topics such as privacy, law, or ethics and no clear link to the EPM literature were excluded.

After applying the above inclusion and exclusion criteria to the identified 161 papers, we excluded 86 papers and therefore 75 papers remained for further analysis.

### Backward and Forward Snowballing

The remaining 75 papers where then used for backward snowballing, which added 83 papers and based on our inclusion and exclusion criteria yielded 118 papers since 40 papers had been removed. To get the most complete picture possible we used those 118 papers for forward snowballing using Google Scholar. This method resulted in 8,147 hits and 89 further papers were investigated and were added to the literature base resulting in a total of 207 papers. After applying our inclusion and exclusion criteria again (which resulted in the removal of 75 papers), we ended up with a total of 132 papers which we grouped into the following paper types: 102 empirical, 12 conceptual, 12 reviews, and six commentary papers. This literature basis of 132 papers is the foundation of our analyses in the following section.

## Epm Framework

**FIGURE 2:**
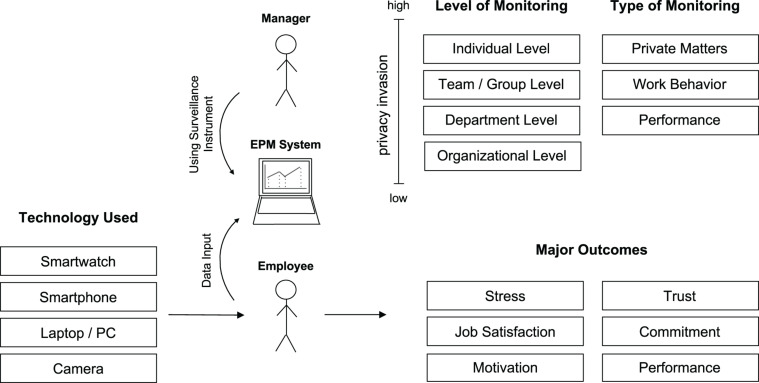
EPM framework.

Figure 2 summarizes our conceptualization of the EPM phenomenon. In the following, we describe the framework.

First, EPM takes place on four levels: individual, team/group, department, and organization (based on Grant and Higgins, 1989). The level of monitoring is correlated with the level of privacy invasion. As indicated in Figure 2, privacy invasion increases from organizational to individual. Regarding the type of monitoring, we distinguish private matters, work behavior, and performance. For example, in a call center it is technologically possible to record the number of an employee’s visits to the bathroom (private matter), his conversations with colleagues (work behavior), and the number of telephone calls and their duration (performance). Moreover, different technologies can be used for monitoring: smartwatch, smartphone, Laptop/PC, and camera. The most important outcomes that have been studied in the literature (for details, see the following sections) are stress, job satisfaction, motivation, trust, commitment, and performance. We use the framework in Figure 2 as basis for the following discussion of the extant literature and for an outline of potential avenues for future research.

### Level of Monitoring

The most abstract level of monitoring is organizational which is characterized by the lowest level of privacy invasion. Monitoring at this level refers to organizational data such as overall achievement of objectives (e.g., number of customers acquired within a certain period of time). The next level is the department level, which breaks down organizational data into the single departments within an organization. Privacy invasion is still low at this level, as inference on individuals is hardly possible (assuming that the number of department members is not too low). Monitoring at the team or group level follows. Teams or project groups can exist within a department or across departments. They consist of a few members only and hence the monitored data can be narrowed down. Therefore, it follows that the privacy invasion level increases. The last level is individual and here the monitored data can be linked to a specific person. Consequently, privacy invasion reaches the maximum.

### Type of Monitoring

We distinguish between three different types of monitoring. First, a company needs to assess performance and this measurement of performance is a precondition for the measurement of goal accomplishment and hence it is frequently part of standardized controlling processes. The second type is work behavior. Here, data is gathered to understand individuals’ work behavior, including human-human, human-task, and human-computer interactions. The major goals of monitoring work behavior are optimization of processes and workflows and user-centered design of information systems. The third type of monitoring refers to private matters of employees by using, for example, location sensing technologies (McNall and Stanton, 2011). Privacy invasion is highest in this case if compared to performance and work behavior monitoring. A well-documented example of surveillance with high privacy invasion is when a company monitors an employee’s private email messages sent via the office PC.

### Technology Used

Different technologies can be used for EPM: cameras (often referred to as closed-circuit television (CCTV) which are also used in public areas), laptops/PCs, the smartphones, and smartwatches (that frequently include various sensors, e.g., motion sensors). The usage of IoT devices expands the general meaning of EPM (Sherif and Al-Hitmi, 2017). Such technologies have the characteristic of being “always-on” which allows the employer to track behavior throughout a whole working day. The use of socio-metric employee badges that use a microphone, a location sensor, or accelerometers, among other technologies, can collect movement and location data of the employee in order to maximize the performance of each individual (Whitmore et al., 2015). According to Gartner, 30% of all organizations worldwide used IoT devices in 2017 and by 2020 this number was expected to rise up to 65% (Hung, 2017). More and more managers also use social media monitoring in order to keep track of employees’ online activities and to make future HR decisions (Elzweig and Peeples, 2009; Suen, 2018). There are also completely new forms of monitoring, such as tracking sleep patterns of employees (Tredinnick and Laybats, 2019) which gives the employer the chance to discover potential well-being issues in order to react before they turn into a serious health problems. However, such data may not be used necessarily in the best interest of employees.

### EPM Outcomes

Despite the fact that employers’ main motivation for using EPM systems use is performance related, EPM may have positive and negative impacts on employees. In this article, we refer to those impacts as outcomes. In the following, we report on major findings of scientific studies which investigated specific outcomes in the EPM context. We summarize our findings in Table 1.

#### Stress

Besides multiple benefits for employees (e.g., the automation of tedious tasks) and organizations as a whole (e.g., reduced cycle times, cost savings, and innovations) that come along with the introduction of ICT in the workplace (Carayon et al., 1999), there are also downsides or negative side effects. One negative aspect is that ICT has become a new source of stress which is referred to as technostress (Riedl, 2013). There is a considerable amount of literature on the effects of EPM on stress. The pervasiveness of technology in the workplace can lead to technostress and to further consequences such as fatigue, burnout, depression, as well as reduced user and job satisfaction (Ayyagari et al., 2011; Riedl et al., 2012; Riedl, 2013; Maier et al., 2015; Tams et al., 2018; Tarafdar et al., 2019; Fischer and Riedl, 2020; Fischer et al., 2021).

Early work carried out by Smith et al. (1992) revealed that employees perceive an increased level of stress when EPM is in use. The survey participants of this study also reported higher job boredom, anxiety, anger, fatigue, health complaints, and psychological tension as a consequence of EPM. Since then experiments on the possible stress effects of EMP were conducted. In essence, experimental research confirmed that EPM may lead to notable stress in employees (Rogers et al., 1990; Carayon, 1994; Hawk, 1994; Varca, 2006). However, despite the evidence presented, studies exist which have only found weak or even no correlation between EPM use and stress (Huston et al., 1993; Nebeker and Tatum, 1993; Bartels and Nordstrom, 2012). In addition, it was found that age moderates the relationship between EPM use and stress. Specifically, older individuals (*M* = 46.9 years) showed a higher stress tendency than younger individuals (*M* = 22.1) (Mallo et al., 2007).

In the 1990s, it had already been argued that further research is critical in this stress domain, as non-significant research findings could eventually be attributed to laboratory situations (Huston et al., 1993; Galletta and Grant, 1995) or sample characteristics of the studies, such as use of students as subjects (Bartels and Nordstrom, 2012). What follows is that more examinations in field settings, as well as studies with non-student samples, are needed. Unfortunately, the call for such studies which date back to the 1990s has not been addressed satisfactorily so far.

#### Motivation

Motivation is a major concept in the work environment (Herzberg et al., 1959) and it denotes a stimulus to accomplish objectives. Evidence for the motivational effects of EPM is inconsistent. There are studies that show a positive effect of EPM use on job motivation, in particular when individual monitoring is applied rather than group monitoring (Aiello and Kolb, 1995; Bartels and Nordstrom, 2012; Gichuhi et al., 2016). However, there are also studies showing that there is a negative effect (O’Donnell et al., 2013) or no effect at all (Rietzschel et al., 2014).

In general, we observe much less evidence of EPM effects on motivation when compared to stress effects. However, the most consistent finding in this domain is that EPM seems to have a positive impact on job motivation, especially in the context of simple and repetitive tasks. However, more research is necessary to arrive at definitive conclusions, in particular with respect to more complex and less repetitive tasks which characterize the work environments of today’s knowledge workers.

#### Job Satisfaction

Job satisfaction is a major determinant of organizational performance (e.g., Bakotić, 2016). Thus, it is a crucial factor from an organizational psychology and business perspective. Evidence indicates that monitored employees are less satisfied with their job than non-monitored ones (Jeske and Santuzzi, 2015). Moreover, it was found that monitoring intensity may decrease job satisfaction but corresponding results were not always statistically significant (Bartels and Nordstrom, 2012; Rietzschel et al., 2014). However, it is also reported that an employee’s possibility to turn off a monitoring system may positively affect job satisfaction (Douthitt and Aiello, 2001). Moreover, Alder and Ambrose (2005) report that EPM positively affects job satisfaction when employees receive positive feedback about the monitoring. Perceived justice, as well as trust, acted as mediators. This study also found that perceived inappropriateness of EPM methods reduced job satisfaction.

A major finding from prior research is that the correlation between monitoring and job satisfaction is more likely to become positive when (i) employees are informed about the monitoring beforehand (Stanton and Barnes-Farrell, 1996) and (ii) employees receive the information that quality aspects of their performance, rather than behavior in general, are recorded (Stanton and Sarkar-Barney, 2003). Despite privacy concerns that employees have which may lead to lower job satisfaction (Seppänen et al., 2015), it is reported that comprehensible reasons for monitoring communicated by the manager to the employees may result in increased satisfaction (Wells et al., 2007). Such reasons may refer, for example, to increased safety through monitoring.

Overall, we found that the relationship between EPM and job satisfaction is moderated by a number of factors including monitoring of qualitative work aspects rather than quantitative ones (Stanton and Julian, 2002) and employees’ perception that the monitoring is a fair measure of the employer (Wells et al., 2007; Zweig and Scott, 2007).

#### Trust

In accordance with seminal academic work (e.g., Rousseau et al., 1998), the Oxford English Dictionary defines trust as “firm belief in the reliability, truth, or ability of someone or something.” In organizations it is desirable that employees trust each other, trust their supervisors and trust the organization in general (e.g., Mayer et al., 1995). Workman (2009) found that employees’ attitude toward EPM was more positive when they had more trust in the organization. Alge et al. (2004) indicate that little trust in employees leads to increased EPM use. Studies found that EPM use may have a negative impact on employee trust toward the organization (Stanton and Sarkar-Barney, 2003; Jensen and Raver, 2012; Holland et al., 2015). However, research also indicates that organizational trust may increase when employees are informed about the monitoring as well as the monitoring purpose in advance (Hovorka-Mead et al., 2002; Alder et al., 2006; McNall and Roch, 2009). In general, however, we observe that in a majority of studies EPM negatively affected trust. Yet, as indicated, it is likely that not the technology itself reduces employees’ trust, or even leads to distrust, but the lack of a transparent organizational communication policy may cause reduced trust and/or increased distrust (Westin, 1992).

#### Commitment

Three different types of commitment have been investigated: organizational commitment, organizational citizenship behavior (OCB) and counterproductive work behavior (Mowday et al., 1979; Jensen and Raver, 2012). Organizational commitment is defined as a behavioral attitude, involving employees’ identification with the organization and implies behaviors that exceed set expectations (Mowday et al., 1979). Several studies show that EPM negatively influences organizational commitment. The stronger the extent of monitoring, the lower this form of commitment (Chang et al., 2015). OCB refers to behavior beneficial to the organization outside employees’ duties (Jensen and Raver, 2012). The results are mixed in this domain. A positive impact of EPM on OCB is reported in one study (Bhave, 2014), a negative impact in other studies (O’Donnell et al., 2013; Jeske and Santuzzi, 2015), and no impact has been reported too (Niehoff and Moorman, 1993; Jensen and Raver, 2012). Counterproductive work behavior denotes deliberate actions with the intention of harming the organization and its stakeholders (Jensen and Raver, 2012). Several authors report a positive correlation between EPM and counterproductive work behavior (Greenberg and Barling, 1999; Wellen et al., 2009; Jensen and Raver, 2012; Martin et al., 2016).

#### Performance

Corporate performance management refers to methodologies, metrics and processes used to manage the business performance of an organization. Considering Zajonc’s (1965) social facilitation theory, one would assume that the mere presence of EPM (used by the supervisor) should result in performance increase as this theory states that improvement in individual performance takes place when working with other people, or being supervised by another person (because a perception of being observed exists; note that this theory predominantly holds true for simple tasks). A series of studies examined the correlation between EPM and performance to verify this assumption.

Irving et al. (1986) were among the first to carry out a study on performance outcomes in the context of EPM. A stable finding in the literature is that the relationship between EPM use and performance is moderated by task difficulty. The relationship is positive when the task is easy and negative when the task is complex or when it involves creativity (Huston et al., 1993; Davidson and Henderson, 2000). Another study by Goomas and Ludwig (2009) showed a direct positive effect of EPM on warehouse workers’ performance, confirming social facilitation theory. However, research also indicates that performance may directly decrease as a consequence of EPM use (Mallo et al., 2007; Becker and Marique, 2014). Importantly, despite the positive and negative performance effects of EPM, there are also studies which show no significant effect at all (Griffith, 1993; Kolb and Aiello, 1996). This leads to the conclusion that no clear-cut statement can be made on the performance effects of EPM. Thus, there is a need for future investigations based on the consideration of other influencing factors and therefore more sophisticated theoretical models.

Table 1 summarizes our findings on the effects of EPM use. The effect of EPM use on the six outcome variables may be positive [+], negative [−], or no effect may exist [∼]. As an example in the stress category, we indicate “Aiello and Kolb (1995) [−].” This indicates a negative EPM effect. Thus, in this study monitored people felt more stressed than non-monitored individuals. The same logic can also be applied to the other outcomes. An implemented EPM system can increase motivation and is therefore labeled with [+] (Aiello and Kolb, 1995), it can reduce job satisfaction [−] (Jeske and Santuzzi, 2015), lead to less trust [−] (Jensen and Raver, 2012), lower organizational commitment [−] (Chang et al., 2015), and increase performance [+] (O’Donnell et al., 2013).

## Moderation Effects

**FIGURE 3:**
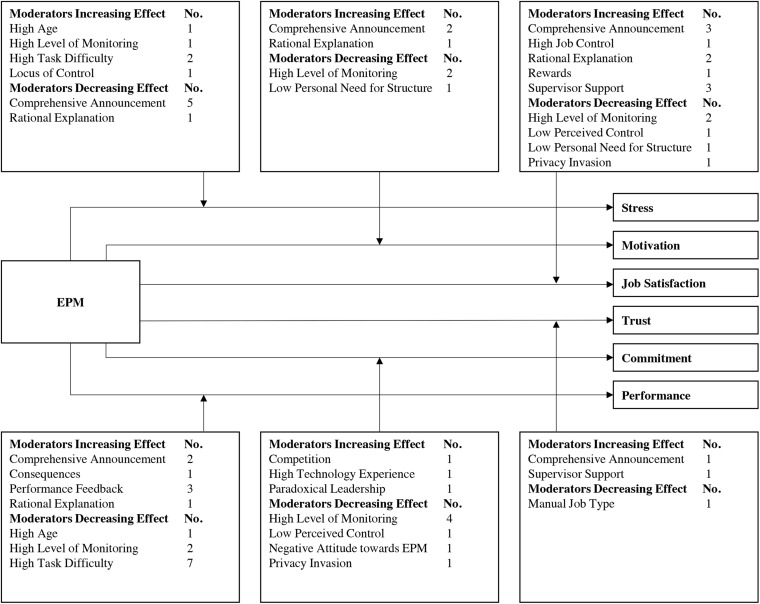
EPM research model with focus on moderation effects.

As already indicated based on example studies in the prior sections, scientific research reveals potential moderating effects in the relationship between EPM use and outcome variables. We systematically analyzed moderation effects and summarized them in Table 1. As an example, the relationship between EPM use and stress is moderated by age. The notation “High Age: Mallo et al. (2007) [↑]” indicates that this specific study found that older individuals are more stressed than younger individuals through EPM use. In addition to Table 1, we summarize the moderation effects in Figure 3 (“No.” in this figure indicates number of identified studies). The six major outcome variables in EPM research are shown on the right side, and all moderation effects which we identified based on our comprehensive literature review are illustrated.

## Privacy Invasion, Ethics, and Culture

According to Westin (1967), “privacy is the claim of individuals, groups, or institutions to determine for themselves when, how and to what extent information about them is communicated to others” (p. 7). An impairment of privacy is referred to as privacy invasion. This form of invasion does not only exist when employees rely on IT equipment in the organization to do their work (Kidwell and Sprague, 2009). Rather, privacy invasion is also a major phenomenon in home office contexts which frequently imply the use of private IT infrastructure. Therefore, recent EPM research (e.g., Ravid et al., 2020) and reports in practice (Satariano, 2020) have picked up this topic.

Based on our literature review, we identified six studies that examined privacy issues in the context of EPM. The first study examined monitoring in the context of technostress by means of a survey. Ayyagari et al. (2011) concluded that the extent of perceived anonymity and thus the inference about one’s own performance, correlates negatively with the perceived violation of privacy. Another study found that identification of an employee through monitoring results in more negative attitudes toward EPM (Carpenter et al., 2016). This result is in line with the finding that invasion of privacy has a negative impact on attitudes toward surveillance (Zweig and Webster, 2002) and that a higher intensity of surveillance leads to a higher perceived invasion of privacy than a lower intensity (O’Donnell et al., 2013). However, the possibility to exert control over surveillance increases perceived data protection and reduces the perceived violation of privacy (McNall and Stanton, 2011). Moreover, if perceived threats of attacks or perceived serious security risks exist, employees’ attitude toward surveillance becomes more positive (Workman, 2009). Next, we complement this summary of scientific findings on EPM and perceptions of privacy invasion with a practical example.

The example we have chosen concerns the storage of all sent emails in a Customer Relationship Management (CRM) system. The emails in such a system comprise personal and organizational messages and all emails are accessible to all authorized employees in the company (e.g., all CRM users). The privacy issue is caused by the fact that also personal messages (e.g., casual messaging with a client or private messages to a person outside the organizational context), are often accessible by a larger group of people. In addition, it is possible that other CRM users manipulate one’s personal messages (e.g., by deleting emails). Importantly, similar situations exist with many other types of organizational application systems (e.g., enterprise resource planning systems or process mining tools).

Even worse from a privacy invasion perspective, monitoring does not necessarily require use of dedicated application systems such as CRM. Rather, surveillance can also take place via (1) messaging software (e.g., instant messaging), (2) communication software (e.g., videoconferencing, intranet, voice over IP), (3) office programs (e.g., word processing, document management), (4) collaboration software (e.g., file sharing), or (5) workplace mobile IT devices software (e.g., smartphone, laptop) (Attaran et al., 2019). Based on these facts, however, one may not conclude that the use of EPM only has negative consequences for privacy. Rather, several ways exist to implement EPM systems in a way to ensure the privacy of employees. Thus, it is possible to leverage the possible benefits of EMP (e.g., increased productivity or safety) and simultaneously avoid its potential drawbacks, particularly perceptions of privacy invasion. Research suggests several procedures to successfully implement EPM. As stated by Chen and Pfleuger (2008) EPM systems create many ethical and privacy concerns about what is being monitored and through what technological means. This leads to the implication that first it should be clear which policies apply to the use of company property such as laptop or smartphone and the general scope of those policies should be transparent as well. Second, employees should be involved in the decision on monitoring policies (Alder and Tompkins, 1997). A study by Jiang et al. (2020) recommends that organizations should inform their employees which information will be subject to monitoring and how their privacy will be protected because such a procedure raises monitoring policy acceptance among personnel. A recent example of EPM that protects employee privacy while providing relevant information to the employer is MS Office 365 (after significant criticism regarding an earlier system version, e.g., *The Guardian*, 2020). It protects employee privacy by *not* providing any usernames and by only delivering aggregated data and not individual data (Microsoft, 2021). Also, it is recommended in the literature to find the “zone of acceptance” as discussed by Stanton and Stam (2006) in which employees do not question the fairness of organizational monitoring (Alder and Tompkins, 1997; Zweig and Webster, 2002). Employees typically feel concerned about their privacy when novel and significant changes appear but usually not because of routine information requests such as timesheets (Stanton and Stam, 2006), workplace communications (Best et al., 2006), or personal information (e.g., age) as they are considered “common practice” (Stanton, 2002). The same applies to information that could also be obtained from other sources (e.g., websites, CV) (Stanton, 2002). Thus, employees in several situations tolerate privacy invasion as offshoot of digital technology advances (Ayyagari et al., 2011).

Regarding data protection it has been suggested anchoring a holistic data protection concept within the organization (Holthaus et al., 2015). This concept is based on two pillars: technical/organizational data protection and strategic/organizational data protection. In the first pillar, the technical implementation of measures by means of data protection concepts or procedure directories is pursued in order to ensure data protection. The second pillar pursues the objective of anchoring data privacy and data security in the organization in the long term through instruments such as employee training or target agreements. Based on the implementation of such a concept, it is intended to achieve a sustainable and holistic anchoring of data privacy throughout the entire organization.

The use of EPM systems at the workplace should always involve ethical considerations because it is related to such topics as stress-related illnesses, fairness judgments, and privacy rights (Rosenberg, 1999). Following ethics conceptualization in philosophy, Alder (1998) differentiates two groups of ethicists in the context of workplace monitoring: teleological and deontological. Teleological ethics refers to a concept of morality that derives moral obligation from what is good, or desirable, as an end to be achieved (Alder, 1998). Frankenna (1973) describes this kind of ethics tellingly: “An act is right if and only if it or the rule under which it falls produces or is intended to produce at least as great a balance of good over evil as any available alternative, an act is wrong if and only if it does not do so” (p. 14). The teleological approach is mostly used by business groups that defend the use of monitoring systems to measure worker performance. In sharp contrast to this view, deontological ethics argues that the morality of an action, or behavior in general, should be based on whether that action or behavior itself is right or wrong (if compared to existing rules), and not based on the consequences of the action or behavior (Frankenna, 1973). The dominant ethics perspective in the EPM literature is that it is more useful to *not* tussle over whether the teleological or deontological is right or wrong; rather, a more useful approach is to focus on how EPM can be designed and used in an ethically sound way.

Another important factor that influences acceptance of EPM use and possible consequences is culture. Culture in general can be defined as a set of shared beliefs, values, and behaviors in social interaction, and organizational culture can be defined as the operating system combining a socially constructed reality including organization’s beliefs, perceptions and values (Alder, 2001). Organizational culture has a significant impact on performance and effectiveness (Deal and Kennedy, 1982; Barney, 1986), predominantly mediated by altered employee attitudes and motivation, as well as by changed individual productivity (Wilkins and Ouchi, 1983; Cooke and Rousseau, 1988; Warrick, 2017).

The most prominent work on culture in societal and organizational contexts was published by Hofstede (1980). In its most recent version, his cultural dimensions theory comprises six dimensions of culture: power distance, individualism, masculinity, uncertainty avoidance, long term orientation, and indulgence. Hofstede’s model has been applied in the EPM context. However, only two out of the 132 reviewed studies directly focus on culture (Alder, 2001; Panina and Aiello, 2005). In essence, these studies report that negative reactions toward EPM can be expected in individualistic cultures, because people in such cultures are more sensitive in terms of privacy invasion if compared to less individualistic cultures (Alder, 2001; Panina and Aiello, 2005). Furthermore, people who live, or work, in a low power distance culture have a tendency to reject EPM as monitoring constitutes signal of distrust, see also Chang et al. (2015). Also, it has been shown that managers in an uncertainty avoidance culture tend to use EPM systems within their organization, because the information generated by EPM reduces uncertainty (Panina and Aiello, 2005). Finally, Alder (2001) found that bureaucratic cultures respond more favorably to monitoring systems in comparison to supportive cultures. Altogether, our analyses show that examinations on EPM use from a cultural perspective holds significant research potential. Despite the few mentioned studies, there is a paucity of corresponding research.

## Future Research Opportunities

The future development of EPM systems is influenced by various trends in practice and science including increased perception of privacy concerns (e.g., Bélanger and Crossler, 2011; Carpenter et al., 2016; Chory et al., 2016), changes in legislation, lifelogging as a behavior to track one’s own life based on digital technologies such as smartwatches (e.g., Weiss et al., 2016; Fischer and Riedl, 2019), altered user behavior to increasingly share private data (e.g., on social media platforms), and technological developments, such as IoT, machine learning, big data and artificial intelligence (e.g., Wenzel and van Quaquebeke, 2017; Ande et al., 2019; Yanqing et al., 2019).

Against the background of these developments, it is likely that EPM adoption will grow substantially in the future. When EPM is applied correctly, it is possible to reduce the potential negative effects such as those reviewed in this article (e.g., increased stress, as well as reduced job satisfaction, motivation, trust, commitment, and performance; see Table 1). However, as indicated in this article, clear-cut research findings on the consequences of EPM use are the exception rather than the rule. What follows is that the relationship between EPM use and outcomes is usually moderated by a range of factors, most of which should be studied more systematically in the future (please refer to Table 1 as a starting point). Therefore, many research opportunities exist. In addition to moderation effects, mediating factors in the relationship between EPM use and outcome variables should be focused on too. This would help to develop a better understanding of *why* and *how* EPM use or specific types of EPM use (e.g., a combination of different levels and types of monitoring along with corresponding technologies, see our framework in Figure 2), may exhibit specific effects or not. As a starting point for answering *why*- and *how*-questions, we documented theories which have been used in extant EPM literature. Based on analysis of the 132 reviewed papers, we identified 14 theories. For a summary, please see the Supplementary Material.

We also emphasize that EPM also offers great research potential for design science scholars. In essence, behavioral insights as presented in this article can be used to build EPM systems. Specifically, a focus on *technological rules* as described by van Aken (2004) could be a viable avenue for future design science research studies. The logic of technological rules can be described as follows (van Aken, 2004, p. 227): *If you want to achieve OUTCOME X, then DESIGN Y will help*. Design does not only refer to system attributes (e.g., features or user interface), but also to organizational context design decisions. An example: If you want to achieve high job satisfaction, then comprehensive announcement of EPM before implementation in the organization will help. Note that employee and supervisor attributes (e.g., their personality), among other factors, may also exert influence on outcome variables.

Designers and engineers should keep in mind that the example only states one design element, namely comprehensive announcement. However, in reality the design of an EPM system would consider a *configuration of factors* and not just one factor. The three design elements of our EPM framework in Figure 2 (used technology, level of monitoring, type of monitoring) and the moderators in Table 1 may serve as a starting point for organizational designers and engineers to build and effectively implement an EPM system.

**FIGURE 4:**
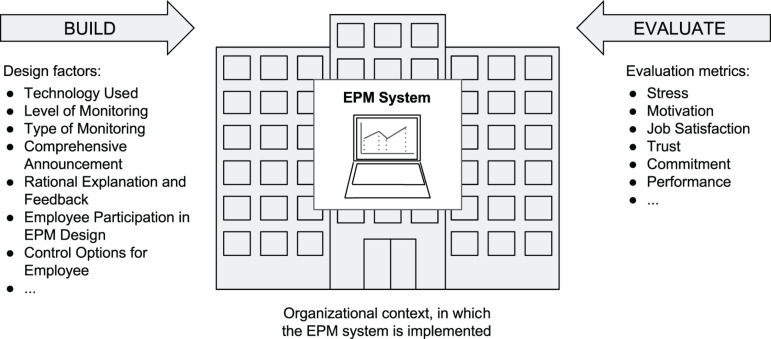
EPM design science framework.

Once an EPM system or different system versions have been developed, the artifact(s) should be evaluated. As indicated by March and Smith (1995), evaluation refers to how well an artifact works, and metrics have to be used to assess the performance, utility, quality, effects, and/or efficacy of the artifact (Walls et al., 1992; Hevner et al., 2004; Gregor and Jones, 2007). The outcome variables in Table 1 (see also Figure 3) may serve as metrics in evaluation studies. Figure 4 outlines our EPM design science framework. The illustration shows the design science research activities “build” and “evaluate” and assigns the three design elements used technology, level of monitoring, and type of monitoring (Figure 2) along with moderator examples (the full list of moderators is shown in Table 1) to the build activity and the six outcome variables to the evaluate activity.

## Concluding Statement

Electronic performance monitoring is a scientific topic which has existed since the 1980s when PCs and ICT became widely introduced in the economy. As a consequence of the recent technological developments related to machine learning, big data, and artificial intelligence, among others, it is not surprising that EPM is experiencing a heyday. Surveillance of the digital workplace, therefore, has recently become a major topic. In this article, we developed a framework to conceptualize EPM (Figure 2), reviewed the scientific literature on six major consequences of EPM use along with moderation effects (Table 1), and outlined how these insights could be used to design and implement EPM systems (Figure 4). Moreover, we discussed possible privacy issues, as well as ethical and cultural considerations. It is hoped that the present work instigates future studies which ultimately lead to EPM systems that are beneficial to both employees and organizations. It will be rewarding to see what insight future research will reveal.

## Author Contributions

RR conceptualized the study and was responsible for funding acquisition. TK reviewed the literature under supervision of RR. TK and RR wrote the manuscript together. Both authors contributed to the article and approved the submitted version.

## Conflict of Interest

The authors declare that the research was conducted in the absence of any commercial or financial relationships that could be construed as a potential conflict of interest.
