# Supplementary material for: Electronic Performance Monitoring in the Digital Workplace: Conceptualization, Review of Effects and Moderators, and Future Research Opportunities
Source: Front Psychol. 2021 May 21;12:633031. doi: 10.3389/fpsyg.2021.633031 (PMC8176029; doi:10.3389/fpsyg.2021.633031)
Supplement: Supplementary file 1 [file Table_1.docx]

Supplementary Material

| *Theory (original source)* | *EPM Literature (referencing the theory)* |
| --- | --- |
| **Agency Theory**  Jensen, M. C., Meckling, W. H. (1976) Theory of the firm: Managerial behavior, agency costs and ownership structure. Journal of Financial Economics 3, 305–360. | Alge, B. J., Ballinger, G. A., Green, S. G. (2004) Remote control: Predictors of electronic monitoring intensity and secrecy. Personnel Psychology 57, 377–410. [Empirical Study] ﻿  “Agency theory is predicated upon the existence of principal-agent relationships in which a principal (e.g., owner) is de- pendent upon an agent (e.g., manager) to perform for the firm.” Alge et al. (2004) p.381 |
| **Balance Theory**  Smith, M. J., Sainfort, P. (1989) A balance theory of job design for stress reduction. International Journal of Industrial Ergonomics 4, 67–79. | Lund, J. (1992) Electronic performance monitoring: A review of research issues. Applied Ergonomics 23, 54–58. [Review Study] ﻿  “Based upon a balance theory (Smith and Sainfort, 1989) perspective, it is recommended that future EPM research should focus on the specification of constructs and measures to assess and control the effects of technology, job task, work environment and organiz- ational structure on individual stress responses.” Lund (1992) p.58 |

| **Deterrence Theory**  Becker, G. S. (1968) Crime and punishment: an economic approach, Journal of Political Economy 76, 169–217. | Chen, J. V., Pfleuger, P. (2008) Employees’ behaviour towards surveillance technology implementation as an information assurance measure in the workplace. International Journal of Management and Enterprise Development 5, 497–511. [Conceptual Study]  “Deterrence Theory (DT) and strategies were used in developing this research construct. Much of DT is concerned with punishment, sanction threats and criminal conduct.” Chen and Pfleuger (2008) p.499 |
| --- | --- |
| **Equity Theory**  Adams, J. C. (1965) Inequity in social exchange. In Berkowitz, L., Ed., Advances in Experimental Social Psychology 2, 267–299. | Hovorka-Mead, A. D., Ross, W. H., Whipple, T., Renchin, M. B. (2002) Watching the detectives: Seasonal student employee reactions to electronic monitoring with and without advance notification. Personnel Psychology 55, 329–362. [Empirical Study]  “[…]is consistent with equity theory, which posits that outcomes are evaluated as fair only in relation to inputs (Adams, 1965).” Hovorka-Mead et al. (2002) p.354 |
| **Ethical Theory**  Beauchamp, T. L., Bowie, N. E. (1993) Ethical Theory and Business - Fourth Edition | Hawk, S. R. (1994) The effects of computerized performance monitoring: An ethical perspective. Journal of Business Ethics 13, 949–957. [Empirical Study] ﻿  “Ethical theory provides the basis for evaluating the morality of a given CPM.” Hawk (1994) p.950 |

| **Expectancy Theory**  Vroom, V. H. (1964) Work and motivation. Malabar, FL: Robert E. Krieger | Nebeker, D. M., Tatum, B. C. (1993) The effects of computer monitoring, standards, and rewards on work performance. Journal of Applied Social Psychology 23, 508–536. [Empirical Study]  “Motivation to perform is determined in part by the expectation that increasing one’s efforts will lead to increases in performance and reward” Nebeker and Tatum (1993) p.534 |
| --- | --- |
| **Feedback Intervention Theory**  Kluger, A. N., DeNisi, A. (1996) The effects of feedback interventions on performance: A historical review, a meta-analysis, and a preliminary feedback intervention theory. Psychological Bulletin 119, 254−284.  ﻿ | Alder, G. S. (2007) Examining the relationship between feedback and performance in a monitored environment: A clarification and extension of feedback intervention theory. Journal of High Technology Management Research 17, 157–174. [Empirical Study]  Alder, G. S., Ambrose, M. L. (2005) Towards understanding fairness judgments associated with computer performance monitoring: An integration of the feedback, justice, and monitoring research. Human Resource Management Review 15, 43–67. [Empirical Study]  “[…] feedback intervention theory (FIT) indicates that feedback recipients may be motivated to focus their attention on task-motivation (e.g., the task itself) or on meta-task processes involving the self. FIT further posits that feedback cues determine which level of action regulation will receive the most attention.” Alder (2007) p.169 |

| **Psychological Reactance Theory**  Brehm, S. S., Brehm, J. (1981) Psychological reactance: A theory of freedom and control. New York: Academic Press. | Martin, A. J., Wellen, J. M., Grimmer, M. R. (2016) An eye on your work: how empowerment affects the relationship between electronic surveillance and counterproductive work behaviours. The International Journal of Human Resource Management 27, 2635-2651. [Empirical Study]  Yost, A. B., Behrend, T. S., Howardson, G., Darrow, J. B., Jensen, J. M. (2019) Reactance to electronic surveillance: a test of antecedents and outcomes. Journal of Business and Psychology 34, 71–86. [Empirical Study]  “Psychological reactance theory provides an understanding of how organizational control mechanisms, such as electronic surveillance, relate to employees’ behavioural ﻿responses.” Martin et al. (2016) pp.2636-2637 |
| --- | --- |
| **Reinforcement Theory**  Skinner, B. E. (1953) Science and human behavior. New York: Macmillan | Alge, B. J., Ballinger, G. A., Green, S. G. (2004) Remote control: Predictors of electronic monitoring intensity and secrecy. Personnel Psychology 57, 377–410. [Empirical Study]  Bartels, L. K., Nordstrom, C. R. (2012) Examining big brother’s purpose for using electronic performance monitoring. Performance Improvement Quarterly 25, 65–77. [Empirical Study] **﻿**  “In terms of reinforcement theory, behavior may be affected by its consequences. Behavior that leads to positive outcomes tends to be repeated, while punishments are avoided.” Bartels and Nordstrom (2012) p.68 |

| **Self-Regulation Theory**  Kanfer, F. H. (1970) Self-regulation: research, issues, and speculation, in C. Neuringer and J.L. Michael (Eds). Behavior Modification in Clinical Psychology, New York: Appleton-Century-Crofts 178–220. | Chen, J. V., Pfleuger, P. (2008) Employees’ behaviour towards surveillance technology implementation as an information assurance measure in the workplace. International Journal of Management and Enterprise Development 5, 497–511. [Conceptual Study] ﻿  “The function of self-regulation theory that pertains most to this study is its use in explaining the cognitive bias known as illusion of control.” Chen and Pfleuger (2008) p.502 |
| --- | --- |
| **Social Facilitation Theory**  Zajonc, R. B. (1965) Social facilitation. Science 149, 269–274. | Aiello, J. R., Douthitt, E. A. (2001) Social facilitation from triplett to electronic performance monitoring. Group Dynamics: Theory, Research, and Practice 5, 163–180. [Conceptual Study]  Aiello, J. R., Kolb, K. J. (1995) Electronic performance monitoring and social context: impact on productivity and stress. Journal of Applied Psychology 80, 339–353. [Empirical Study]  Aiello, J. R., Svec, C. M. (1993) Computer monitoring of work performance: Extending the social facilitation framework to electronic presence. Journal of Applied Social Psychology 23, 537–548. [Empirical Study]  Chen, J., Ross, W. H. (2005) The managerial decision to implement electronic surveillance at work: A research framework. International Journal of Organizational Analysis 13, 244–268. [Conceptual Study]  Stanton, J. M., Sarkar-Barney, S. T. M. (2003) A detailed analysis of task performance with and without computer monitoring. International Journal of Human-Computer Interaction 16, 345–366. [Empirical Study] **﻿**  “Social facilitation theory deals with the impact of social presence on individual performance. […] ﻿Social facilitation theory now refers not only to performance enhancements, but also to impairments.” Aiello and Douthitt (2001) p.163 |
| **Social Information Processing Theory**  Salancik, G. R., Pfeffer, J. (1978) A social information processing approach to job attitudes and task design. Administrative Science Quarterly 23, 223–253. | Stanton, J. M., Julian, A. L. (2002) The impact of electronic monitoring on quality and quantity of performance. Computers in Human Behavior 18, 85–101. [Empirical Study]  “SIP theory predicts that the social environment has profound influences on employees’ attitudes and behaviours by providing a variety of cues that workers interpret to make sense of the environment and act in concert with its demands.” Stanton and Julian (2002) p.88 |
| **Theory of Planned Behaviour**  Ajzen, I. (1985) From intentions to actions: A theory of planned behaviour, in J. Kuhl and J. Beckmann (Eds). Action-Control: From Cognition to Behavior, New York: Springer 11–39. | Chen, J. V., Pfleuger, P. (2008) Employees’ behaviour towards surveillance technology implementation as an information assurance measure in the workplace. International Journal of Management and Enterprise Development 5, 497–511. [Conceptual Study]  Spitzmüller, C., Stanton, J. M. (2006) Examining employee compliance with organizational surveillance and monitoring. Journal of Occupational and Organizational Psychology 79, 245–272. [Empirical Study]  ﻿  “Ajzen (1991; Ajzen & Madden, 1986) developed the theory of planned behaviour, which has subsequently proved useful for the prediction of organizational behaviour based on attitudes, beliefs, social norms, intentions, and volitional control.” Spitzmüller and Stanton (2006) p.247 |
| **Virtue Theory**  Aristotle (1980) The Nicomachean Ethics. (D. Ross, Trans.) Oxford: Oxford University Press.  MacIntyre, A. (1984). After virtue: A study in moral theory (2nd ed.). South Bend, IN: University of Notre Dame Press. | West, J. P., Bowman, J. S. (2016) Electronic surveillance at work: An ethical analysis. Administration and Society 48, 628–651. [Conceptual Study]  “Yet, virtue theory’s strength - subjective judgments inferred from personal character - is also its shortcoming: If advocates and opponents of surveillance perceive they are good, they can be convinced that what they do is good.” West and Bowman (2016) p.14 |

Adams, J. C. (1965) Inequity in social exchange. In Berkowitz, L., Ed., Advances in Experimental Social Psychology 2, 267–299.

Aiello, J. R., Douthitt, E. A. (2001) Social facilitation from triplett to electronic performance monitoring. Group Dynamics: Theory, Research, and Practice 5, 163–180.

Aiello, J. R., Kolb, K. J. (1995) Electronic performance monitoring and social context: impact on productivity and stress. Journal of Applied Psychology 80, 339–353.

Aiello, J. R., Svec, C. M. (1993) Computer monitoring of work performance: Extending the social facilitation framework to electronic presence. Journal of Applied Social Psychology 23, 537–548.

Ajzen, I. (1985) From intentions to actions: A theory of planned behaviour, in J. Kuhl and J. Beckmann (Eds). Action-Control: From Cognition to Behavior, New York: Springer 11–39.

Alder, G. S. (2007) Examining the relationship between feedback and performance in a monitored environment: A clarification and extension of feedback intervention theory. Journal of High Technology Management Research 17, 157–174.

Alder, G. S., Ambrose, M. L. (2005) Towards understanding fairness judgments associated with computer performance monitoring: An integration of the feedback, justice, and monitoring research. Human Resource Management Review 15, 43–67.

Alge, B. J., Ballinger, G. A., Green, S. G. (2004) Remote control: Predictors of electronic monitoring intensity and secrecy. Personnel Psychology 57, 377–410.

Aristotle (1980) The Nicomachean Ethics. (D. Ross, Trans.) Oxford: Oxford University Press.

Bartels, L. K., Nordstrom, C. R. (2012) Examining big brother’s purpose for using electronic performance monitoring. Performance Improvement Quarterly 25, 65–77.

Beauchamp, T. L., Bowie, N. E. (1993) Ethical Theory and Business - Fourth Edition.

Becker, G. S. (1968) Crime and punishment: an economic approach, Journal of Political Economy 76, 169–217.

Brehm, S. S., Brehm, J. (1981) Psychological reactance: A theory of freedom and control. New York: Academic Press.

Chen, J. V., Pfleuger, P. (2008) Employees’ behaviour towards surveillance technology implementation as an information assurance measure in the workplace. International Journal of Management and Enterprise Development 5, 497–511.

Chen, J., Ross, W. H. (2005) The managerial decision to implement electronic surveillance at work: A research framework. International Journal of Organizational Analysis 13, 244–268.

Hawk, S. R. (1994) The effects of computerized performance monitoring: An ethical perspective. Journal of Business Ethics 13, 949–957.

Hovorka-Mead, A. D., Ross, W. H., Whipple, T., Renchin, M. B. (2002) Watching the detectives: Seasonal student employee reactions to electronic monitoring with and without advance notification. Personnel Psychology 55, 329–362.

Jensen, M. C., Meckling, W. H. (1976) Theory of the firm: Managerial behavior, agency costs and ownership structure. Journal of Financial Economics 3, 305–360.

Kanfer, F. H. (1970) Self-regulation: research, issues, and speculation, in C. Neuringer and J.L. Michael (Eds). Behavior Modification in Clinical Psychology, New York: Appleton-Century-Crofts 178–220.

Kluger, A. N., DeNisi, A. (1996) The effects of feedback interventions on performance: A historical review, a meta-analysis, and a preliminary feedback intervention theory. Psychological Bulletin 119, 254−284.

Lund, J. (1992) Electronic performance monitoring: A review of research issues. Applied Ergonomics 23, 54–58.

MacIntyre, A. (1984). After virtue: A study in moral theory (2nd ed.). South Bend, IN: University of Notre Dame Press.

Martin, A. J., Wellen, J. M., Grimmer, M. R. (2016) An eye on your work: how empowerment affects the relationship between electronic surveillance and counterproductive work behaviours. The International Journal of Human Resource Management 27, 2635-2651.

Nebeker, D. M., Tatum, B. C. (1993) The effects of computer monitoring, standards, and rewards on work performance. Journal of Applied Social Psychology 23, 508–536.

Salancik, G. R., Pfeffer, J. (1978) A social information processing approach to job attitudes and task design. Administrative Science Quarterly 23, 223–253.

Skinner, B. E. (1953) Science and human behavior. New York: Macmillan

Smith, M. J., Sainfort, P. (1989) A balance theory of job design for stress reduction. International Journal of Industrial Ergonomics 4, 67–79.

Spitzmüller, C., Stanton, J. M. (2006) Examining employee compliance with organizational surveillance and monitoring. Journal of Occupational and Organizational Psychology 79, 245–272.

Stanton, J. M., Julian, A. L. (2002) The impact of electronic monitoring on quality and quantity of performance. Computers in Human Behavior 18, 85–101.

Stanton, J. M., Sarkar-Barney, S. T. M. (2003) A detailed analysis of task performance with and without computer monitoring. International Journal of Human-Computer Interaction 16, 345–366.

Vroom, V. H. (1964) Work and motivation. Malabar, FL: Robert E. Krieger

West, J. P., Bowman, J. S. (2016) Electronic surveillance at work: An ethical analysis. Administration and Society 48, 628–651.

Yost, A. B., Behrend, T. S., Howardson, G., Darrow, J. B., Jensen, J. M. (2019) Reactance to electronic surveillance: a test of antecedents and outcomes. Journal of Business and Psychology 34, 71–86.

Zajonc, R. B. (1965) Social facilitation. Science 149, 269–274.
